# Comparative analysis of dinoflagellate chloroplast genomes reveals rRNA and tRNA genes

**DOI:** 10.1186/1471-2164-7-297

**Published:** 2006-11-23

**Authors:** Adrian C Barbrook, Nicole Santucci, Lindsey J Plenderleith, Roger G Hiller, Christopher J Howe

**Affiliations:** 1 Department of Biochemistry, University of Cambridge, Downing Site, Tennis Court Road, Cambridge, CB2 1QW, UK; 2 Children's Medical Research Institute, 214 Hawkesbury Road, Westmead, Sydney, NSW 2145, Australia; 3 Department of Biological Sciences, Macquarie University, Sydney, NSW 2109, Australia

## Abstract

**Background:**

Peridinin-containing dinoflagellates have a highly reduced chloroplast genome, which is unlike that found in other chloroplast containing organisms. Genome reduction appears to be the result of extensive transfer of genes to the nuclear genome. Unusually the genes believed to be remaining in the chloroplast genome are found on small DNA 'minicircles'. In this study we present a comparison of sets of minicircle sequences from three dinoflagellate species.

**Results:**

PCR was used to amplify several minicircles from *Amphidinium carterae *so that a homologous set of gene-containing minicircles was available for *Amphidinium carterae *and *Amphidinium operculatum*, two apparently closely related peridinin-containing dinoflagellates. We compared the sequences of these minicircles to determine the content and characteristics of their chloroplast genomes. We also made comparisons with minicircles which had been obtained from *Heterocapsa triquetra*, another peridinin-containing dinoflagellate. These *in silico *comparisons have revealed several genetic features which were not apparent in single species analyses. The features include further protein coding genes, unusual rRNA genes, which we show are transcribed, and the first examples of tRNA genes from peridinin-containing dinoflagellate chloroplast genomes.

**Conclusion:**

Comparative analysis of minicircle sequences has allowed us to identify previously unrecognised features of dinoflagellate chloroplast genomes, including additional protein and RNA genes. The chloroplast rRNA gene sequences are radically different from those in other organisms, and in many ways resemble the rRNA genes found in some highly reduced mitochondrial genomes. The retention of certain tRNA genes in the dinoflagellate chloroplast genome has important implications for models of chloroplast-mitochondrion interaction.

## Background

The organisation of the chloroplast genome in many peridinin-containing dinoflagellates has been shown to be very unusual [1-5]. A massive reduction in the gene content of the organelle genome is observed in these organisms relative to all other photosynthetic chloroplasts [6]. EST data from a number of dinoflagellate species suggest that many genes that are typically located within the chloroplast genome have been transferred to the nuclear genome [7-10]. Furthermore, the genome is unusual in that the remaining chloroplast genes are confined to small circular DNA molecules (minicircles) of between 2–10 kb (although larger molecules have been reported in some species [11]), rather than a single large circularly mapping molecule [12]. The minicircles found in dinoflagellates each typically contain a single gene, though up to three genes have been recorded on one minicircle [13]. An interesting feature of these minicircles is the presence of a non-coding region that is well conserved in all gene-containing minicircles of a given species, as well as in 'empty' minicircles which have no obvious gene sequences [1,3,13-15]. However, even within genera there is little or no conservation of DNA sequence identity within this common non-coding region [16]. In contrast, the coding regions of minicircles show high levels of identity within genera. Some controversy exists as to the location of the minicircles. Several indirect lines of evidence support their location in the chloroplast [5]. These include an absence of sequences encoding transit peptides, the localisation of *psbA *transcripts to chloroplasts [17] and chloramphenicol inhibition of PsbA translation [18]. However, a report from one species indicates a possible location of minicircles in the nuclear compartment [4]. This contradiction is not yet resolved, and it remains possible that different dinoflagellate species have circular DNA molecules present in different compartments.

We have characterised what appear to be the complete chloroplast coding genomes of both *A. carterae *and *A. operculatum *as well as a number of the related 'empty' minicircles from each species. It seems likely that few, if any, minicircles remain to be discovered that contain typically chloroplast located genes, since the EST data [7-10] contain examples of almost all the genes which are invariably found on chloroplast genomes that have not been found on minicircles. This provides the basis for a comparative analysis of two sets of minicircles. We have also made comparisons with the other extensively characterised dinoflagellate minicircle set from *Heterocapsa triquetra *where appropriate. Comparative genome analyses are useful in identifying genetic features that may not be apparent from single genome analyses [19,20]. We were particularly interested in examining whether any previously unrecognized genes were present on the minicircles. Genes could have been missed from previous analyses of minicircular sequences, especially if the genes were short or poorly conserved. As the rate of substitution in minicircle genes appears to be high this is a significant concern and similarity searches against sequence databases may have missed genes [2,21]. However, we would expect DNA sequences containing such genes to be conserved between two closely related organisms, such as the two *Amphidinium *species. We also used comparisons between more distantly related genera to help establish the extent of ribosomal RNA genes as the identification of rRNA genes has proved controversial [6]. Other pattern based search algorithms, such as tRNA-scan-SE [22], were used to examine the minicircles for significant genetic features.

The results of these analyses suggest that at least three extra protein-coding regions may be present. We also found the first evidence for tRNA genes on minicircles. We have also further characterised rDNA sequences from minicircles. These sequences, which are transcribed, are highly divergent showing evidence of a high rate of mutation, as well as a possible fragmentation of the gene sequence. The peridinin-containing dinoflagellate rDNA sequences share similarities to the reduced rDNA sequences found in mitochondrial genomes.

## Results and Discussion

### PCR amplification of *A. carterae *minicircles

A DNA fraction from *A. carterae *which had been previously shown to be enriched in minicircles was used as a template for PCR [3]. Fragments of the *A. carterae *genes for *psbB*, *petD *and *atpB *were initially obtained by PCR using degenerate primers based on the corresponding translated gene sequences from *A. operculatum *and 'universal' primers to the core region (Table 1). It was assumed that the gene orientation in *A. carterae *is conserved with respect to the core region. Part of a *psbC *gene sequence was generated by chance from primers CD26f1 and UR (CD26f1 was a primer to reverse of *psbE *but had 10 out of 11 3' bases in common with *psbC*). Full sequences of each minicircle were generated by adjacent opposed specific primers designed according to the fragment sequences. DNA sequencing revealed that all of the minicircle sequences obtained possessed a core region of the type previously described for *A. carterae *[3].

Another minicircle was obtained by PCR with primers designed on the basis of the *A. carterae *core region (UF and UR) only (ecac27: acc. no. DQ507216). A similar approach had been used to obtain nine other empty minicircles [3]. With the characterisation of the four gene-coding minicircles from *A. carterae *(*petD*, *atpB*, *psbC *and *psbB*: acc. nos DQ507217–DQ507220 respectively), we now possessed a homologous set of gene-coding minicircle sequences for the two *Amphidinium *species.

### Overall genome characteristics

**Figure 1:**
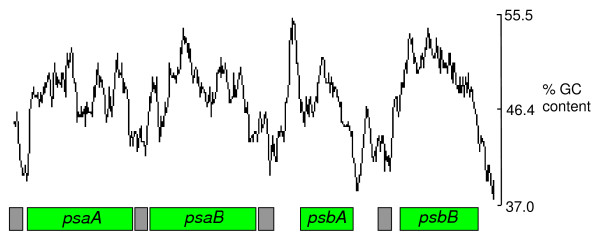
**GC content for the *psaA-*, *psaB-*, *psbA- *and *psbB*-containing minicircles of *A. operculatum***. GC content is calculated within a window of 200 nucleotides. Boxes indicate the positions of genes (green) and core regions (gray).

A summary of the genome sizes and previously annotated gene content is shown in Table 2. The mean GC contents of the *Amphidinium *species minicircles are 45.27% for *A. carterae *and 46.46% for *A. operculatum*. The coding regions generally appear to be more GC-rich than the non-coding regions, as shown by a plot of GC content (Figure 1). The GC content of all of the core regions in both *Amphidinium *species is lower than the overall GC content of the minicircles. Intriguingly the *psbA *gene in both *Amphidinium *species is flanked by a region of unusually high GC content preceding the gene and low GC content after the gene. The mean GC content of *H. triquetra *minicircles is 37.02%, which is much lower than in the *Amphidinium *species. Some of the discrepancy is due to the longer non-coding regions found in the minicircles of *H. triquetra*, as these regions are AT-rich. However, this is not the sole cause. The coding regions of *H. triquetra*, whilst more GC-rich than the core regions, are considerably more AT-rich than the coding regions of *Amphidinium*. This is reflected in the codon usage of the two genera. Codons ending in A or T are more prevalent in *H. triquetra *[2]. The total length of all minicircle sequences (both gene-containing and 'empty') obtained from *A. carterae *is 45,815 bp and from *A. operculatum *is 34,186 bp. The main cause of the difference in lengths is the discovery of many more empty minicircles in *A. carterae *(10 empty minicircles compared to 5 in *A. operculatum*). However, in addition the *A. carterae *minicircles are slightly larger than their *A. operculatum *equivalents, with just a single exception (the *petB*/*atpA *minicircle).

### Comparison of previously identified genes

Coding regions on previously reported minicircles of *A. operculatum *and *A. carterae *had been identified by combination of BLAST searches and CodonPreference analysis of the DNA sequences. The 12 predicted gene-encoding regions in each species all have obvious identity to known chloroplast encoded proteins. The coding regions of *A. operculatum *and *A. carterae *minicircles show high levels of identity at both the DNA and predicted amino acid level (Table 3). The PsaB sequences (beta subunit of Photosystem I) had the lowest identity between species, 97.6%. All other inferred protein sequences have at least 99% identity or above. Therefore, on the basis of their protein sequences it seems that the two dinoflagellate species are closely related. (In addition ultrastructural studies suggest that the *A. operculatum *strain might be more appropriately designated as *A. carterae *{E. Nash, pers. comm.}) The shortest previously identified coding region is for PsbI, a component of Photosystem II, the sequence corresponding to protein of only 35 amino acids [13].

Little difference exists in the codon usage of the two *Amphidinium *species. Marked preferences exist for certain codons for many amino acid residues (data not shown). For example the GGT codon is by far the most frequently used codon for glycine. Other features of the codon usage in *A. operculatum *and *H. triquetra *have been discussed previously [2,23].

An identical set of eleven codons is very infrequently used in both species (10 examples or fewer of each out of 4453 codons). They are TTA (Leu), TCA (Ser), TGA (Stop), CCC (Pro), CGC (Arg), CGG (Arg), ATA (Ile), ACG (Thr), AAA (Lys), AGA (Arg) and GGG (Gly). The frequency of the rare codons is unevenly distributed amongst the minicircle genes. The two genes for the core components of Photosystem I, *psaA *and *psaB*, have higher frequencies of these codons than the other genes as shown in Table 4.

In addition to the previously identified protein genes BLAST searches identified a region with clear identity to a plastid-type LSU rDNA in each species of *Amphidinium*. However, the LSU rDNA sequence does not appear to be a full-length sequence, as will be discussed later. No SSU rRNA gene was identified in an initial search in the *Amphidinium *sequences.

### Further protein genes

**Figure 2:**
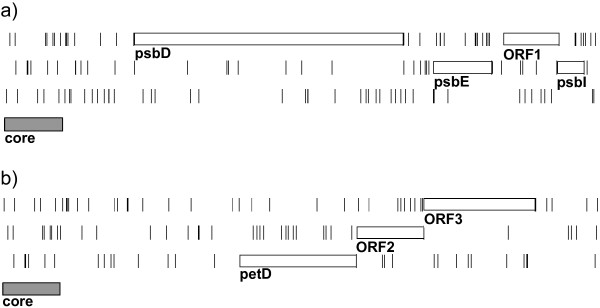
**Positions of additional gene-coding regions**. a) *psbDEI *minicircle of *A. operculatum*. A single further gene is proposed between the *psbE *and *psbI *genes. b) *petD *minicircle of *A. operculatum*. Two further genes are proposed following the *petD *gene. Diagrams show all three reading frames and the positions of all stop codons (vertical lines). Boxes indicate the positions of the proposed protein-coding regions and also the position of the core region on each minicircle. Minicircles were linearised for graphical display by breaking the minicircle immediately prior to the core region.

Artemis and ACT analyses were used to identify regions of high identity between the *Amphidinium *species that were comparable in sequence identity to previously characterised genes (>95%). Using Artemis ORFs within these regions were identified and investigated [see Additional files 1, 2, 3 and 4]. These comparative analyses suggest there may be a further three protein coding regions on the gene-coding minicircles; one more on the *psbD*/*E*/*I *minicircle and two more on the *petD *minicircle. The positions of these putative protein-coding regions are shown in Figure 2. The inferred amino acid sequences have relatively few of the 'rare' codons, as determined from the previously identified genes, though the occurrence of these codons is more frequent (Table 4). None of the putative amino acid sequences gave significant hits in BLAST and FASTA searches of protein databases. One of the sequences (the second ORF on the *petD *minicircle, ORF3) was suggested by FUGUE [24] to encode a ribosomal protein (Rpl15), although the assignment was tentative. An alignment of the ORF3 sequence with other Rpl15 sequences shows a very low level of identity with these sequences. A longer sequence, open apart from a single TAA termination codon, is present in a different reading frame. However, this did not give significant hits in BLAST or FASTA sequences. Furthermore, no evidence of editing has yet been found in *Amphidinium *[15], so we identify the most likely ORF as that shown in Figure 2b. Several other short ORFs are present in areas of minicircles that are neither established coding regions nor core regions. However, these ORFs are either not conserved between the *Amphidinium *species or have high levels of rare codons, and do not give significant hits in protein similarity searches.

Within the *A. operculatum *empty minicircles there is only a single ORF capable of producing a protein of over 100 amino acids on the expected strand in all the 'empty' circles. Six ORFs exist that could produce proteins of at least 75 amino acids. Numerous ORFs exist of comparable size to the *psbI *ORF. Within the *A. carterae *empty minicircles, where more 'empty' minicircles have been identified, three ORFs capable of producing a protein of over 100 amino acids are present on the expected strand together with a further eleven ORFs capable of producing proteins of at least 75 amino acids. However, none of these ORFs of over 75 amino acids is found in their entirety on an empty circle in both species. In some cases short stretches of sequence corresponding to part of these ORFs show high levels of identity (>90%) between the species. However, in all these cases either the level of identity rapidly falls or frame shifts are introduced in one of the sequences.

One of the ORFs found only in *A. carterae *is of note in that it is predicted by the FUGUE search algorithm to be a ribosomal protein (Rps3) gene. The gene for this protein is invariably found in the plastid genome of all other plastid-containing organisms. So far the gene for this protein has not been found in any of the dinoflagellate EST projects, although it should be noted that these projects are not comprehensive with regard to plastid targeted gene sequences. Alignments with other Rps3 sequences are not conclusive in identifying the ORF. They suggest that the first domain of the protein, if it is an Rps3, is truncated.

### RNA genes

Typically chloroplast genomes encode a number of important functional RNA molecules. These include tRNAs, rRNAs and in some taxa tmRNA, the RNA component of RNase P and the RNA associated with the SRP-like protein. We carried out sensitive searches of regions of high identity (>90%) between species to identify whether such components are encoded in the dinoflagellate chloroplast genome [see Additional files 1, 2, 3, 4, 5, and 6]. For the larger RNA molecules we attempted to establish their organisation and extent [see Additional files 7, 8, 9 and 10]. Typically this was achieved by using Bestfit to identify matches to short conserved nucleotide motifs that are found in the functional RNAs. Regions identified by this approach were checked against multiple alignments. Surrounding sequences were analysed to see if there was potential for forming appropriate secondary structures [see Additional files 11 and 12]. This was achieved using a combination of visual inspection and the *M*fold program. In generating assignments we made extensive use of structure models, especially those of Gutell *et al*. for rRNAs [25].

### Ribosomal RNA

**Figure 3:**
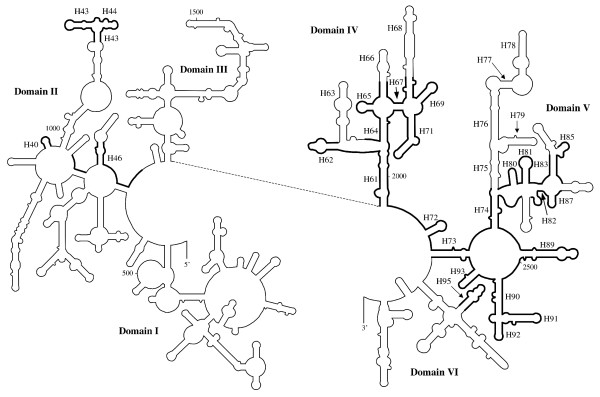
**Schematic diagram indicating the extent of the LSU rRNA gene in *Amphidinium***. Structural model of the folding of the 5' and 3' halves of LSU rRNA from *E. coli*. Regions where homologous sequence or structure is found on *Amphidinium *minicircles are in bold. The proposed base-pairing of the individual structures is displayed in additional file 11, along with detailed numbering of the positions of the structures. Helices proposed to be present in *Amphidinium *or that are mentioned in the text are labelled (e.g. H18 for helix 18). Helix numbering as defined by Ban *et al*.[26].

Within the *Amphidinium *species only one sequence with significant identity to a functional RNA has been previously identified [3,13], showing similarity to a LSU rRNA gene. However, based on similarity searches this did not appear to be a full-length LSU rDNA sequence. We studied the sequence further to establish the probable size of the LSU ribosomal sequence and whether the sequence conforms to structural models of other chloroplast LSU ribosomal RNAs [25]. This comparison revealed that stretches of nucleotides sharing identity to conserved regions of other chloroplast LSU rDNAs are found only for domains II, IV, V and VI on the LSU rDNA minicircles of *A. carterae *and *A. operculatum *[26]. Domains I and III appear to be either missing or so divergent that alignment with other LSU rRNA molecules proves impossible. Even the sequences which lie within the domains II, IV, V and VI are highly unusual compared with LSU rRNA genes from other chloroplasts. A higher substitution rate is apparent, and there is frequent deletion or truncation of helical elements (see Figure 3).

Only short stretches of domain II can be assigned. Many of the short stretches correspond to loop regions between helices (see Figure 3). The most notable feature that can be identified comprises helices 43 and 44. These helices are RNA components of the 'stalk', which is known to interact with elongation factors [26].

Domain IV is the most strongly conserved domain found. However, significant truncations of the sequence are clearly discernible. Helix 63 appears to have been completely lost. This is accompanied by a shortening of the following loop. Helix 66 appears to have been significantly modified and helix 68 is much shorter than is typical. Despite the *Amphidinium *sequences sharing fewer identities with other chloroplast ribosomal sequences than is usual, the overall folding of the molecule seems to be maintained.

Sequences corresponding to domain V are clearly discernible for both *Amphidinium *species. However, numerous truncations or mutations appear to have altered the capability of forming a typical structure. The truncations are almost exclusively found in regions corresponding to stem-loop structures, rather than the loop regions between stem-loops (Figure 3). In particular truncation of the region corresponding to helices 75–79 appears to be very extensive and an alternative folding is predicted that does not resemble more typical models. The nature of the sequence corresponding to domain V, in terms of mutations and truncations, is similar to those described by Santos *et al. *in their study of domain V of LSU rDNA of the genus *Symbiodinium *[27].

The only feature of domain VI that can be assigned is the sarcin/ricin loop (helix 95). Identity to other LSU rDNA sequences break downs soon after this feature, and it is possible that this is where the functional sequence ends. It should be noted that the non-core sequence of *A. operculatum *microcircle 1 (415 bp) [13] corresponds almost exactly to the 23S rRNA minicircle sequence after the end of domain V, including the region corresponding to the sarcin/ricin loop.

**Figure 4:**
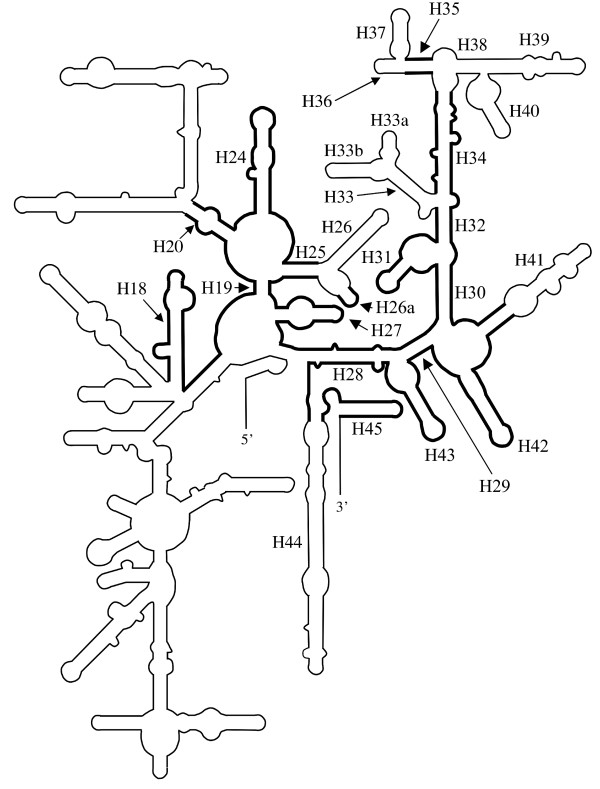
**Schematic diagram indicating the extent of the LSU rRNA gene in *Amphidinium***. Structural model of the folding of the SSU rRNA from *E. coli*. Regions where homologous sequence or structure is found on *Amphidinium *minicircles are in bold. The proposed base-pairing of the individual structures is displayed in additional file 12, along with detailed numbering of the positions of the structures. Helices proposed to be present in *Amphidinium *or that are mentioned in the text are labelled (e.g. H95 for helix 95). Helix numbering as defined by Wimberley *et al. *[28].

**Figure 5:**
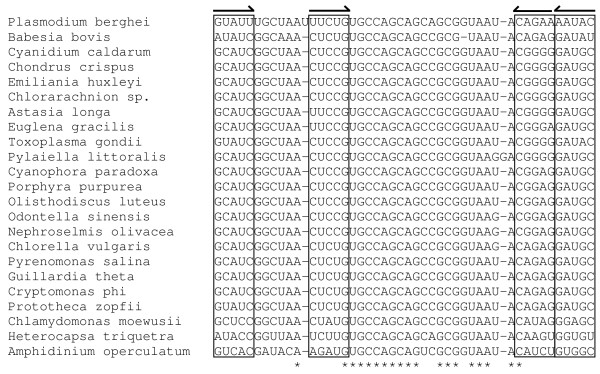
**Alignment of chloroplast SSU rDNA sequences corresponding to helix 18**. Boxes and arrows indicate regions of complementary base pairing. Asterisks indicate positions of nucleotide identity.

Short SSU rRNA sequences have previously been reported from empty circle 4, but they were believed to be non-functional owing to their length [15]. The surrounding sequences showed very low identity to other predicted SSU rDNAs; the first block of SSU rRNA sequence we identified is helix 18 (Figure 4). This feature contains the highly conserved 530 loop that is involved in proofreading of the mRNA/tRNA interaction [28]. The loop region itself shows high levels of identity with all other SSU rRNA sequences as shown in Figure 5. However, the sequence of the stem either side of the loop is very divergent with respect to other chloroplast SSU rRNAs (Figure 5). Indeed within this feature it appears to be the most divergent of all known chloroplast sequences. Despite the incorporation of these base changes base-pairing within the stem loop structure is maintained, suggesting selective evolutionary pressure is still present.

We found that there is a much larger intervening sequence between two of the elements that we identified (the 5' and 3' strands of helix 20 [Figure 4]) than is usually the case, 902 nucleotides rather than the 165 nucleotides (positions 588–753 *E. coli *[Figure 4]) that would be normally expected. None of the intervening sequences in *Amphidinium *resembled features typically found in SSU rRNAs. This suggests that the sequences preceding and following these elements could be transcribed separately or that an intron could be present.

The second block of SSU rRNA sequence we identified is much longer than the first and comprises sequences corresponding to positions 754–1542 (3' end) of the *E. coli *sequence. Despite having very low levels of identity to other SSU rDNAs the sequence is capable of folding to form most of the secondary structure elements found in such molecules. Some peripheral features do appear to have been lost or truncated, namely regions corresponding to helices 26, 33, 33a, 33b, 36, 37, 38, 39, 40 and 44 (see Figure 4).

We have found no evidence for a 5S rRNA gene.

The highly divergent nature of the LSU and SSU rDNA sequences raises the probability that they are pseudogenes rather than functional sequences. Clearly the rDNA sequences are unlike any that have been previously described from chloroplast genomes. Even the sequences from the apicoplasts of sporozoa, such as *Toxoplasma gondii*, whilst showing high levels of substitution have retained essentially all the structural features, including all the domains, found in other plastid rDNAs [25]. The closest example to the sequences found on dinoflagellate minicircles comes from highly derived mitochondrial genomes. In many mitochondrial rDNAs there are extensive examples of deletions and truncations of many structural elements including entire domains, as well as examples of fragmented rDNA sequences. In the most reduced examples peripheral features are extensively deleted whilst key regions which contribute to essential features such as the A, P and E sites are retained [29]. Our analyses suggests that this is what we find with regard to the dinoflagellate rDNA sequences. Nucleotide positions that are known to contribute to the A, P and E sites are generally well conserved in *Amphidinium *as well as other important features such as proof-reading and decoding sites. It is also possible that other rDNA fragments exist that "fill in" missing parts of the molecules. Thus the rRNAs could be assembled from separate bits, as has been found elsewhere (e.g. *Chlamydomonas *mitochondria [30]). The molecules could either remain separate or be joined together by *trans*-splicing.

**Figure 6:**
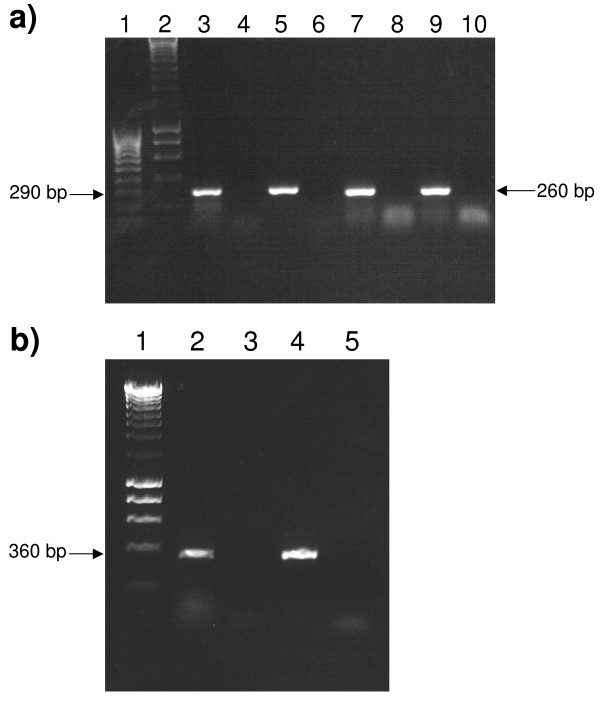
**RT-PCR analysis of LSU and SSU rRNA sequences**. a) Lane 1, Hyperladder IV markers (Bioline); Lane 2, Hyperladder I markers (Bioline); Lane 3, RT-PCR with primers LSU1F and LSU2R and *A. operculatum *RNA template; Lane 4, as Lane 3 but no RT enzyme; Lane 5, PCR with primers LSU1F and LSU2R and *A. operculatum *DNA template; Lane 6, as Lane 5 but no DNA template added; Lane 7, RT-PCR with primers LSU3F and LSU4R and *A. operculatum *RNA template; Lane 8, as Lane 7 but no RT enzyme; Lane 9, PCR with primers LSU3F and LSU4R and *A. operculatum *DNA template; Lane 10, as Lane 9 but no DNA template added. b) Lane 1, Hyperladder I markers (Bioline); Lane 2, RT-PCR with primers SSU1F and SSU2R and *A. operculatum *RNA template; Lane 3, as Lane 2 but no RT enzyme; Lane 4, PCR with primers SSU1F and SSU2R and *A. operculatum *DNA template; Lane 5, as Lane 4 but no DNA template added.

We therefore carried out RT-PCR of representative regions of LSU and SSU rRNA. Using specific primers we amplified two regions for the predicted LSU rRNA; these corresponded to sequences from 3' of helix 46 to 5' of helix 62 and from 5' of helix 62 to 5' of helix 72. For the SSU rRNA gene we amplified a region from 3' of helix 27 to 5' of helix 43. Precise primer positions are specified in the supplementary data files BMCGenLSU.tab and BMCGenSSU.tab respectively. For both RNAs products were obtained of sizes corresponding to genomic DNA (Figure 6), whose sequences were also consistent with the genomic DNA. These initial data indicate that the putative rRNA genes are transcribed and remain essentially unmodified.

### tRNA

**Figure 7:**
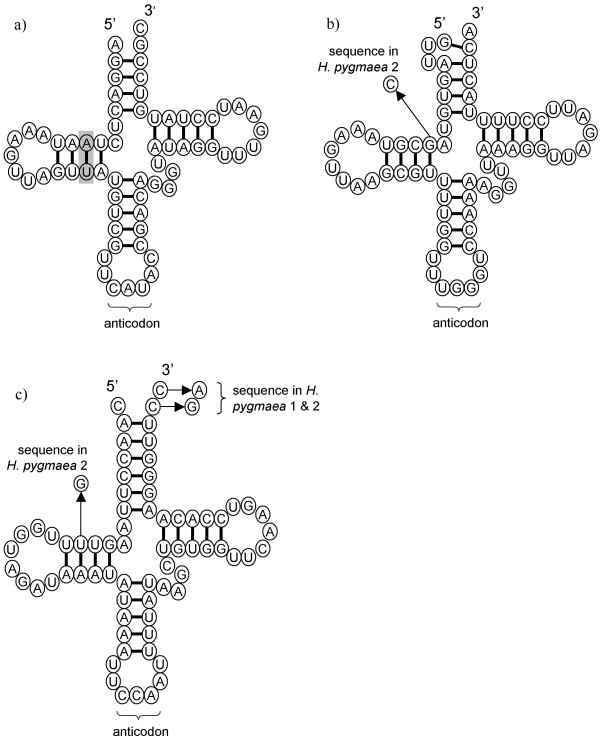
**Structural diagrams of tRNA sequences found on minicircles of peridinin-containing dinoflagellates**. Sequence variation between species or different copies within a species is indicated. Structures are as suggested by tRNAscan-SE v.1.2. a) Structure of the tRNA^fMet ^encoded on empty circle 4 (ecao4: AF401630) in the dinoflagellate *Amphidinium operculatum*. The purine:pyrimidine base pair in the dihydrouridine stem characteristic of fMet-tRNAs is highlighted [31]. b) Structure of the tRNA^Pro ^encoded on plastid minicircles in the dinoflagellates *H. triquetra *and *H. pygmaea *(*H. pygmaea *1: AF206707, *H. pygmaea *2: AY033400). c) Structure of the tRNA^Trp ^encoded on plastid minicircles in the dinoflagellates *H. triquetra *and *H. pygmaea*.

Searches for tRNAs in *Amphidinium *revealed a single putative example, which is present in both species. The sequence suggests that it is a formyl-methionine initiator tRNA, as there is an absence of Watson-Crick base pairing at the end of the acceptor stem and also there is a characteristic purine:pyrimidine base pair in the dihydrouridine stem, in contrast to a pyrimidine:purine base pair which is found in other tRNAs [31]. The predicted structure of the *A. operculatum *tRNA is shown in Figure 7a. The *trnfM *sequence is found adjacent to the 3' end of the core region on empty minicircle 4 in *A. operculatum *and empty minicircle 33 in *A. carterae *(both of which we now believe to contain the unusual SSU rRNA gene) and is almost completely identical between the two species. It may be significant that it is a *trnfM *sequence that is retained as no equivalent tRNA species exists in the cytosol which could be imported as a replacement [32]. Many organelles with highly reduced genomes lacking a full complement of tRNA genes are believed to import cytosolic tRNAs to maintain translation within the organelle [33].

A homologous fMet-tRNA was not found in any of the *Heterocapsa *species sequences, although two other putative tRNA sequences were found, one for Pro-tRNA and one for Trp-tRNA (Figure 7b, 7c respectively). In *H. triquetra *both putative tRNA sequences are found on minicircles that do not have full-length gene sequences, but have truncated versions of at least two other genes ('jumbled' minicircles) [14]. One such circle carries a single tRNA gene, whilst in three others the two tRNAs are found in tandem. All of the tRNA sequences found on each of the different 'jumbled' minicircles are identical. In *Heterocapsa pygmaea *these two same tRNA sequences are found in tandem on *psbA *minicircles, almost immediately after the *psbA *coding region. Two distinct *psbA*-containing minicircles have been isolated from *H. pygmaea*, and both contain the tRNA sequences. The tRNA sequences are almost identical to the *H. triquetra *sequences (Figure 7b, 6c). Some sequence variation exists between the tRNA sequences on each of the minicircle in *H. pygmaea*. In one of the tandem tRNA copies (*H. pygmaea *2) this variation disrupts base-pairing in the tRNA structures (Figure 7b, 7c). As there are apparently at least two copies of the gene it is possible that one of the sequences is redundant and is no longer under selective pressure.

### Other RNA species

Searches for other RNA species that have previously been discovered in other chloroplast genomes did not yield any significant matches. Thus we found no evidence for RNase P, tmRNA or SRP-associated RNA.

## Conclusion

The acquisition of complementary sets of minicircles from two *Amphidinium *species has facilitated the identification of several genetic features on the minicircles that had not previously been recognised. We suggest that a further three protein coding genes are present on the minicircular chloroplast genome of both *A. operculatum *and *A. carterae*. These genes appear do not bear similarity to typically chloroplast genome located genes. They may therefore be specific to dinoflagellates and could be connected to the unusual genome organisation. Evidence from transcripts levels in *A. carterae *suggests that these open reading frames are expressed at levels comparable to other genes that have been found on minicircles such as *psbD *(R. Hiller, in preparation).

We have also been able to locate a partial SSU rRNA gene. This was found on what had previously been described as empty minicircles in both *Amphidinium *species. With the exception of one further minicircle from *A. carterae *we have not found genes on any of the other empty minicircles, though their presence cannot yet be ruled out. It is possible that editing might restore presently unrecognized coding sequences. Although editing has been reported from *C. horridum*, no evidence has been found for it in *Amphidinium*, although only a limited number of transcripts have been tested [15]. We determined the extent of the both the SSU rRNA and LSU rRNA genes by sequence and folding similarity to other chloroplast genes. This revealed the extremely unusual nature of these genes. Numerous features of the chloroplast rRNA molecules are missing from these sequences, including whole domains in the case of the LSU rDNA. It is possible that these domains could be transcribed from a distinct DNA locus and the rRNA reassembled post-transcriptionally. However, the RT-PCR data suggest that this is not the case. Further transcript analysis will be needed to confirm this, but it seems that the extent and architecture of the *Amphidinium *sequences most closely resembles the severely truncated rDNAs found in some mitochondrial genomes, and represents the most divergent chloroplast rDNAs yet found.

We also report the discovery of the first tRNA genes to be found on minicircles. These appear to be very limited in number and it is therefore likely that the peridinin-containing chloroplast is reliant on the import of cytosolic tRNAs for chloroplast translation. It is interesting that the only tRNA to be found so far in the *Amphidinium *species is an fMet-tRNA for which a cytosolic counterpart does not exist. It has been suggested that the plastid provides fMet-tRNA for the mitochondrion in Apicomplexa [32]. Although no complete dinoflagellate mitochondrial genome sequence has yet been published, no tRNA genes have been identified in the partial sequences available at present [34]. Given this, we suggest that the dinoflagellate plastid likewise supplies fMet-tRNA to the mitochondrion.

Our analyses further highlight the unusual nature of the peridinin-containing dinoflagellate chloroplast genome, which is characterised by highly reduced gene content, atypical genomic organisation and highly divergent gene sequences. However, the existence of divergent genes sequences may have lead us to underestimate the genetic capacity of the minicircular genomes, when they are examined in isolation. Comparative analyses of the dinoflagellate genomes, particularly closely related genomes, appear to be a useful tool in identifying significant features. Based on our analyses of the *Amphidinium *genomes the minicircles may be more densely packed with genes than we thought. Further comparative analyses of other dinoflagellate chloroplast genomes are likely to be useful.

## Methods

### Culture Conditions

*A. carterae *CS21 was cultured under continuous illumination (20 μEinsteins.m^-2^·s^-1^) at 18°C in Provasoli's enriched sea water. *A. operculatum *(from the Culture Collection of Algae and Protozoa, Oban, UK, ref CCAP 1102/6) was cultured under a 16 h light (25 μEinsteins.m^-2^·s^-1^)/8 h dark cycle at 21°C in f/2 media.

### DNA Isolation, PCR amplification and cloning of minicircular sequences

Template DNA for PCR was obtained from total DNA from *A. carterae *as described by Hiller[3]. Primers used in PCR reactions are described in Table 1. Standard PCR conditions were an initial cycle of 94°C for 1 minutes followed by 35 cycles of 94°C for 1 minutes, 52°C for 1 minutes, 72°C for 4 minutes. PCR products were cloned into pGEM-T plasmid vector (Promega) and transformed into *Escherichia coli *prior to sequencing.

### DNA Sequencing and Computational Analysis of Sequences

DNA clones were sequenced using the automatic dye terminator system (ABI 377). BLAST analyses were used to identify conserved chloroplast genes. Minicircle DNA sequences were assembled and analyzed using the GCG Wisconsin package (version 11.1, Accelrys Inc., San Diego, CA). The Bestfit, Compare, Dotplot and Gap programs, which are part of the GCG Wisconsin package, were used to identify regions of identity between minicircle sequences.

### Accession numbers of sequences used

*Amphidinium operculatum *sequences used were: *psaA *[EMBL:AJ250264]; *psaB *[EMBL:AJ582639]; *psbA *[EMBL:AJ250262]; *psbB *[EMBL:AJ250263]; *psbC *[GenBank:AF426172]; *psbD*/*E*/*I *[EMBL:AJ620761]; *petB*/*atpA *[GenBank:AY048664]; *petD *[EMBL:AJ250265]; *atpB *[EMBL:AJ250266]; LSU rRNA [EMBL:AJ582640]; ecao4 (SSU rRNA) [GenBank:AF401630]; ecao1 (empty circle *A. operculatum *1) [GenBank:AF401627]; ecao2 [GenBank:AF401628]; ecao3 [GenBank:AF401629] and ecao5 [EMBL:AJ582641].

*Amphidinium carterae *sequences used were: *psaA *[EMBL:AJ311631]; *psaB *[EMBL:AJ311629]; *psbA *[EMBL:AJ311632]; *psbB *[Genbank:DQ507216]; *psbC *[GenBank:DQ507219]; *psbD*/*E*/*I *[EMBL:AJ311628]; *petB*/*atpA *[EMBL:AJ311630]; *petD *[GenBank:DQ507217]; *atpB *[GenBank:DQ507218]; LSU rRNA [EMBL:AJ311633]; ecac33 (SSU rRNA) [EMBL:AJ318067]; ecac2 (empty circle *A. carterae *2) [EMBL:AJ307009]; ecac10 [EMBL:AJ307010]; ecac11 [EMBL:AJ307011]; ecac14 [EMBL:AJ307012]; ecac15 [EMBL:AJ307014]; ecac17 [EMBL:AJ307013]; ecac25 [EMBL:AJ307015]; ecac27 [GenBank:DQ507216] and ecac82 [EMBL:AJ307016].

*Heterocapsa triquetra *sequences used were: *psaA *[GenBank:AF130031]; *psaB *[GenBank:AF130032]; *psbA *[GenBank:AF130033]; *psbB *[GenBank:AF130034]; *psbC *[GenBank:AF130035]; *petB *[GenBank:AF130037]; *atpA *[GenBank:AF130036]; LSU rRNA [GenBank:AF130039]; SSU rRNA [GenBank:AF130038]; abc1 (aberrant circle 1) [GenBank:AY004267]; abc2 [GenBank:AY004268]; abc3 [GenBank:AY004269]; abc4 [GenBank:AY004270] and abc5 [GenBank:AY004271].

*Heterocapsa pygmaea *sequences used were: *psbA *[GenBank:AF206707] and *psbA2 *[GenBank:AY033400].

### Artemis and ACT analysis

Artemis and Artemis Comparison Tool (ACT) were used for whole genome analyses of minicircle sequences [35,36]. For these analyses minicircle sequences were concatenated as linear DNA sequences. The circular sequences were linearised by breaking immediately 5' of the core region. The sequence and annotation files for use with Artemis are available as additional files in this publication [See Additional files 1, 2, 3, 4, 5, 6, 7, 8, 9, 10]. ACT was used to visualise regions of identity between species. Regions of identity were determined by pairwise Blast [37]. The output of the pairwise Blast was then used as an input into ACT.

### RNA searches

Identification of potential structures of rRNA was facilitated by comparison of the dinoflagellate sequences to structural models of rRNAs. These were obtained from the Comparative RNA website [25]. Potential folding of inferred RNA sequences was explored using *M*fold [38]. Searches for potential tRNA sequences were carried out with tRNAscan-SE v.1.2 using a mitochondrial/chloroplast source model [22]. The tmRNA website was used for similarity searches between a database of tmRNAs and the dinoflagellate sequences [39].

### RNA extraction and RT-PCR analysis

Template RNA for reverse transcriptase reactions was obtained from *A. operculatum *cells using the RNeasy Mini Kit (QIAGEN) according to manufacturer's instructions. Total RNA was subsequently incubated with RQ RNase-free DNase (Promega) for 1 hour at 37°C, after which 5 μl STOP solution was added and the DNase inactivated by incubation at 65°C for 10 minutes. For first-strand DNA synthesis 5 μl RNA preparation was mixed with 20 pmol of the relevant RT primer in a total volume of 15 μl. This mixture was incubated at 70°C for 5 minutes then rapidly cooled on ice. To this initial volume 5 μl of Moloney-Murine Leukemia Virus RT reaction buffer (Promega), 1.25 μl of dNTPs (10 mM each), 25 U of RNasin (Promega) and 200 U of Moloney-Murine Leukemia Virus reverse transcriptase (M-MLV RT) were added and the reaction mixture brought to 25 μl with nuclease-free water. The reverse transcription reaction was incubated at 42°C for 1 hour. Controls with no M-MLV RT added were also performed. Subsequent PCR was carried out using 5 μl of the reverse transcription reaction mixture to which 25 μl MasterAmp 2× PCR Premix A (Epicentre Technologies), 2 μl 25 mM MgCl_2_, 25 pmol of each primer and 1.25 U GoTaq DNA polymerase (Promega) were added and the reaction mixture brought to 50 μl. Standard PCR conditions were an initial cycle of 95°C for 2 minutes followed by 35 cycles of 95°C for 1 minute, 52°C for 1 minute, 72°C for 1 minute and a final cycle of 72°C for 10 minutes. Positive controls which included *A. operculatum *total DNA instead of M-MLV RT reaction mix and negative controls with no template addition were also carried out as well as the no M-MLV RT control described above. PCR products obtained were cloned into pGEM-T Easy plasmid vector (Promega) and transformed into *Escherichia coli *prior to sequencing.

## Authors' contributions

ACB helped conceive the study, carried out all the *in silico *analyses and drafted the manuscript. NS and RH jointly carried out PCR amplifications of minicircle genes from *A. carterae *and sequenced the products. LJP carried out the RT-PCR experiments. CJH helped conceive the study, participated in its direction and helped draft the manuscript. All authors read and approved the final manuscript.

## Supplementary Material

Additional File 1 ***A. carterae *Artemis sequence file**. Plain text file containing concatenated *A. carterae *minicircle sequences for viewing with Artemis a freely available genome viewer and annotation tool. Click here for file

Additional File 2 ***A. operculatum *Artemis sequence file**. Plain text file containing concatenated *A. operculatum *minicircle sequences for viewing with Artemis a freely available genome viewer and annotation tool. Click here for file

Additional File 3 ***H. triquetra*Artemis sequence file**. Plain text file containing concatenated *H. triquetra *minicircle sequences for viewing with Artemis a freely available genome viewer and annotation tool. Click here for file

Additional File 4 ***A. operculatum *Artemis annotation file**. Artemis annotation file for use with additional file 1. Contains gene location information. Click here for file

Additional File 5 ***A. operculatum *Artemis annotation file**. Artemis annotation file for use with additional file 2. Contains gene location information. Click here for file

Additional File 6 ***H. triquetra *Artemis annotation file**. Artemis annotation file for use with additional file 3. Contains gene location information. Click here for file

Additional File 7 ***A. operculatum *LSU minicircle Artemis sequence file**. Plain text file containing *A. operculatum *LSU rRNA minicircle sequence for viewing with Artemis, a freely available genome viewer and annotation tool. Click here for file

Additional File 8 ***A. operculatum *LSU minicircle Artemis annotation file**. Artemis annotation file for use with additional file 7. Contains predicted locations of LSU rRNA structures (helices and loops) found on the *A. operculatum *LSU rRNA minicircle. Click here for file

Additional File 9 ***A. operculatum *SSU minicircle Artemis sequence file**. Plain text file containing *A. operculatum *SSU rRNA minicircle sequence for viewing with Artemis, a freely available genome viewer and annotation tool. Click here for file

Additional File 10 ***A. operculatum *SSU minicircle Artemis annotation file**. Artemis annotation file for use with additional file 9. Contains predicted locations of SSU rRNA structures (helices and loops) found on the *A. operculatum *SSU rRNA minicircle. Click here for file

Additional File 11 ***A. operculatum *LSU rRNA structures**. Word document containing proposed RNA structures found on the *A. operculatum *LSU rRNA minicircle with detailed base-pairing and numbering. Click here for file

Additional File 12 ***A. operculatum *SSU rRNA structures**. Word document containing proposed RNA structures found on the *A. operculatum *SSU rRNA minicircle with detailed base-pairing and numbering. Click here for file

## Figures and Tables

**Table 1:** Primers used for PCR and RT-PCR.

Primer	Sequence	Amino acid sequence or adjacent rRNA structure
UF	5'-TTTGGAATCTCAGCTCGATTC	
UR	5'-CTCGCACCCAAATTGACT	
CD26f1	5'-gtGATgagGTCTGtAGTGG	
NIC*psbB*	5'-GCNTTYTGGCAYTGGGC	A F W H W A
*petD*f3	5'-GARCCNGCNTGGCCNAA	E P A W P N
*atpB*r1	5'-TCNCGGAARTAYTCNGCC	(M) A E Y F R D (in reverse)
LSU1F	5'-AGTGATACTGTTCTCTTGAG	3' helix 46 LSU rRNA
LSU2R	5'-GGTTTCATAACTCGGTTGTC	5' helix 62 LSU rRNA
LSU3F	5'-GACAACCGAGTTATGAAACC	5' helix 62 LSU rRNA
LSU4R	5'-ACGTCCAGTGTCATTTCACC	5' helix 72 LSU rRNA
SSU1F	5'-GAAACTTAAAGGTGCTGGTGG	3' helix 27 SSU rRNA
SSU2R	5'-GTTACTAACGATTCCAGCTTC	5' helix 43 SSU rRNA

Primers used to amplify initial fragments of *psbC, psbB, petD *and *atpB *genes from *A. carterae *and primers used in RT-PCR analyses of the *A. operculatum *rRNAs. UF (universal forward) and UR (universal reverse) are adjacent opposed primers made to the core region. In CD26f1 the mismatches with *psbC *sequence are shown in lower case.

**Table 2:** Type and size of minicircle sequences in the chloroplast genomes studied

	*A. carterae*	*A. operculatum*	*H. triquetra*
total length (bp)	45,815	34,186	42,769
mean GC content	45.27%	46.46%	37.02%
gene containing minicircles			
*psaA*	2,558	2,443	3,005
*psaB*	2,366	2,363	3,121
*psbA*	2,520	2,311	2,151
*psbB*	2,327	2,282	2,286
*psbC*	2,477	2,341	2,330
*psbD*/*E*/*I*	2,358	2,369	
*psbD*			2,628^#^
*psbE*			2,196^#^
*petB*/*atpA*	2,606	2,713	
*petB*			2,204
*atpA*			2,444
*petD*	2,563	2,416	2,177^#^
*atpB*	2,587	2,483	-
LSU rRNA	2,713	2,651	3,027
SSU rRNA	(2,533)	(2,458)	2,563
'empty' minicircles			
No.	10	5	1
total length of empty minicircles	20,729 (18,196^$^)	9,825 (7,367^$^)	2,012^#^
chimeric minicircles			5
total length of chimeric minicircles			10,625

^$^Not including putative SSU rRNA minicircle.

^#^These sequences were submitted to the sequence database whilst this paper was in submission, the sequences do not therefore form part of our comparative analysis.

**Table 3:** Percentage identities of minicircle ORFs

	*A. operculatum*	*H. triquetra*
	percentage identities	percentage identities

PsaA	99.3	50.7
PsaB	97.6	42.7
PsbA	99.7	86.1
PsbB	100.0	59.9
PsbC	99.6	64.5
PsbD	99.7	na
PsbE	100.0	na
PsbI	100.0	na
PetB	99.1	75.3
PetD	100.0	na
AtpA	99.8	54.1
AtpB	99.3	na
ORF1	98.6	na
ORF2	100.0	na
ORF3	100.0	na
LSU rRNA	92.0 over 2662 base stretch, not all of this may be LSU rDNA. (DNA comparison)	67.6 over 692 base stretch. (DNA comparison)
SSU rRNA	81.1 over 2403 base stretch, not all of this may be SSU rDNA. (DNA comparison)	69.8 over 288 base stretch. (DNA comparison)

Percentage identities were calculated by comparison to *A. carterae *sequences using pairwise Blast [36].

na – sequences unavailable or not found in the other species.

**Table 4:** Distribution of rare codons in *Amphidinium *minicircle protein genes.

	A. c./no.	A. c./total amino acids	% rare codons	A. o./no.	A. o./total amino acids	% rare codons
*psaA*	16	678	2.4	13	671	1.9
*psaB*	9	644	1.4	12	685	1.8
*psbA*	4	340	1.2	2	340	0.6
*psbB*	3	506	0.6	3	506	0.6
*psbC*	5	463	1.1	5	463	1.1
*psbD*	2	355	0.6	1	355	0.3
*psbE*	0	77	-	0	77	-
*psbI*	2	35	5.7	1	35	2.9
*petB*	4	219	0.9	2	219	0.9
*petD*	2	157	1.3	2	157	1.3
*atpA*	4	488	0.8	4	488	0.8
*atpB*	5	590	0.8	6	590	1.0
ORF1 (*psbD*/*E*/*I*)	3	73	4.1	3	73	4.1
ORF2 (*petD*)	5	90	5.6	5	90	5.6
ORF3 (*petD*)	9	150	6.0	8	150	5.3

Rare codons comprise 1.2% of all codons in the previously identified gene set.

A. c.: *Amphidinium carterae*, A. o.: *Amphidinium operculatum*.
