# Supplementary material for: Comparative analysis of dinoflagellate chloroplast genomes reveals rRNA and tRNA genes
Source: BMC Genomics. 2006 Nov 23;7:297. doi: 10.1186/1471-2164-7-297 (PMC1679814; doi:10.1186/1471-2164-7-297)
Supplement: Additional File 11 — A. operculatum LSU rRNA structures. Word document containing proposed RNA structures found on the A. operculatum LSU rRNA minicircle with detailed base-pairing and numbering. [file 1471-2164-7-297-S11.doc]

LSU rRNA - *A. operculatum* – numbering as found in BMCGenLSU.dna

Helix 40

Key

C G : canonical base pair

G U : G-U base pair

G A : G-A base pair

U U : non-canonical base pair

Only G-A and non-canonical base pairs found in other sequences are displayed.

: RNA backbone (where necesaary

GC

GC

GC

C

C

A

A

A

A

1178 -

- 1189

Helix 42, 43 & 44

U

A

U

G

A U

C

C

U

A

U

A

G

A

C

C

C U U

G A A

U G C

A C G

G G U

U C A

C

A

C

A

C

AU

UA

UA

CG

CG

U

C U

U

G

U

A

A

C

1254 -

- 1311

Helix 46 - truncated

A

U G

U G

C G

C A

U U

C G

C G

C G

A U

U A

C G

A

U

C

1377 -

- 1407

C

G

G

U

Helix 61

A

A

U

U

A

U

A

1650 -

- 1913

A U

G C

A U

U A

G C

C G

G C

G A

U A

C G

G U

G U

U A

A

U

C

1669 -

- 1896

Helix 62

U

A

A

G G A U A A U

C A U A U U G

- 1707

A

- 1684

C

U

G

G

A

A

Helix 64

G C

C G

G C

A U

A A

C G

U A

A U

C G

1724 -

- 1886

A

1715 -

- 1895

Helix 65

A C A

U G U

A

C

U

A

A

A

A

U

A

- 1739

- 1725

Helix 66 - truncated

G T

G C

A T

T G

G C

T G

G C

G C

1742 -

- 1774

1749 -

- 1767

Helix 67

C A U G

A U A C

1777 -

1878 -

- 1780

- 1875

Helix 68 - truncated

U G

U A

C G

G U

U A

A U

G C

U A

G C

A

A

A

C

U

A

A

A

C

1781 -

- 1808

Helix 69

1809 -

- 1827

U A

G C

G C

C G

U G

G C

U

G

C

A

U

A

A

Helix 71

C A

G C

A U

C G

U A

G C

U

A

A

A

U

1848-

- 1864

Helix 72

G

A

A

A

G C

C G

A U

U G

G C

1927 -

- 1944

U

U

G

U

Helix 73

1946 -

2447 -

- 1960

- 2433

A

C

U U G G G A A C U G A A A

G U C C C U U A U C U A U

A U

Helix 74

U A

U A

C G

G U

A U

1978 -

- 2257

1967 -

- 2269

C A

U A

C G

C G

A

G C

G C

C

A

Helix 80

U G

U A

A U

G C

2088 -

- 2100

G

G

G

G

C

Helix 81

A

U

A

2101 -

- 2127

C A

G U

U A

C G

C G

A U

C

C

U

C

A

U

U

- 2123

Helices 82, 83, 85 & 87

A

A

G

A

A

G

U

G

G

2165 -

U

A

2136 -

G

A

A

A

G C

G C

U A

A U

G C

G C

A

A

A

A

U

2125 -

A C

U G

C G

G C

A A

C

U

G

A

A

C

U

U

C

A

2229 -

A

U

- 2188

- 2210

G

A

Boxes with lines indicated base pairing between the boxed nucleosides.

Helix 89

2278 -

2319 -

U

C

G

A

U

A

G C U A A U G G G A A A A

C G G U U G C C C U U U A

U

C

U

C

C

A

G

U

A

U

Helices 90, 91 & 92

G C

U G

C G

G C

A U

A U

U A

G C

G C

A U

A U

C G

C G

U A

C G

A G

G

2404 -

- 2330

G

C

C

A

U

U

U U C C A G A G A G

G G G C U C G G

C

A

C

C

G

A

U

U

A

U

A

A

A

C

G

G

G

G

Helix 93

2410 -

2428 -

G

G A A C G A U

U U U G C U A

U

A

A

A

Helix 95 (Sarcin – Ricin loop)

2465 -

- 2127

C G

U A

A U

U A

G C

C

U

C

U

C

A

G

A

A

G

- 2489

G

G

C

A

A
