# Supplementary material for: Comparative analysis of dinoflagellate chloroplast genomes reveals rRNA and tRNA genes
Source: BMC Genomics. 2006 Nov 23;7:297. doi: 10.1186/1471-2164-7-297 (PMC1679814; doi:10.1186/1471-2164-7-297)
Supplement: Additional File 12 — A. operculatum SSU rRNA structures. Word document containing proposed RNA structures found on the A. operculatum SSU rRNA minicircle with detailed base-pairing and numbering. [file 1471-2164-7-297-S12.doc]

SSU rRNA - *A. operculatum* – numbering as found in BMCGenLSU.dna

Helix 18

Key

G C

U A

A U

G C

A U

C G

A U

C G

U G

G C

C G

G C

C

G

C

U

C

A

A

A

A

U

G

G

U

A

U

G

A

A

C

U

A

G

C G : canonical base pair

G U : G-U base pair

G A : G-A base pair

U U : non-canonical base pair

Only G-A and non-canonical base pairs found in other sequences are displayed.

: RNA backbone (where necesaary

746 -

- 791

Helix 19

U A

A U

G C

815 -

- 1845

813 -

- 1847

Helix 20

AU

- 1734

- 832

A G G C

U C U G

U

A

G G C

C C G

823 -

1743 -

C

C

Helix 24

CG

GU

GC

GC

UA

CA

G

U

C

A

A

G

G

U

A

U

G

U

A

G

GC

UA

GC

UA

CG

CA

A

A

U

A

1748 -

- 1789

Helix 25

G G C U A A

U C G A U U

1800 -

- 1805

1843 -

- 1837

G

Helix 26a

These bases pair with a GU sequence found at the beginning of the 570 loop.

A

A

C

A

C G

A U

1820 -

- 1827

Helix 27

GC

CU

AU

CG

GG

UG

GU

U

G

G

G

C

C

A

A

A

A

G

U

A

1850 -

- 1876

Helix 28

G

G A

1885 -

2278 -

- 1901

- 2262

U G G U G U A A U U U A A C

A C U A C G U U G A A U A G

A G

U

Helix29

1903 -

2225 -

- 1907

- 2221

A C G G U

U G C U A

Helix 30, 31 & 32

1955 -

- 2109

U

C

C

G

A

A

C

U

A

C

A

CGA

GCU

U

U

G

U A

A U

G C

U A

A U

G C

U A

C G

A U

A U

G A

G C

U A

A U

G C

C G

A U

C G

C G

G

C

A

G

U

U

A

U

A

G

A

U

U

1908 -

- 2133

Helix 34

2017 -

- 2090

U G

C G

U U

G C

U A

C G

G U

A

C

A

G U

U A

U

A

G U

U G

U G

G C

G C

G C

C

U

2000 -

- 2108

Helix 35

G G U G A U

C C A C U A

2021 -

- 2026

2045 -

- 2040

Helix 41

2143 -

- 2176

U A

G C

G U

U A

C G

C G

A U

2137 -

- 2182

Helix 42

A

A

G

A

U

U

U G

G C

G C

C C

G C

A U

G C

U U

G

A

G

A

A

A

2186 -

- 2215

C G

Helix 43

G A

C C

A U

U A

C G

C G

C G

A U

G

U

U

G

U

A

U

A

U

2231 -

- 2255

Helix 45

2368 -

2391 -

G

U A G G U U A A A C

G U U C A A G U U G

G

A

A
